# The Frequency-Dependent Neuronal Length Constant in Transcranial Magnetic Stimulation

**DOI:** 10.3389/fncel.2016.00194

**Published:** 2016-08-09

**Authors:** Risto J. Ilmoniemi, Hanna Mäki, Jukka Saari, Ricardo Salvador, Pedro C. Miranda

**Affiliations:** ^1^Department of Neuroscience and Biomedical Engineering, Aalto University School of ScienceEspoo, Finland; ^2^A.I.R Advanced Analytics for IoT, Comptel CorporationHelsinki, Finland; ^3^Faculdade de Ciências, Institute of Biophysics and Biomedical Engineering, Universidade de LisboaLisbon, Portugal

**Keywords:** transcranial magnetic stimulation, TMS, length constant, cable equation, membrane potential

## Abstract

**Background:** The behavior of the dendritic or axonal membrane voltage due to transcranial magnetic stimulation (TMS) is often modeled with the one-dimensional cable equation. For the cable equation, a length constant λ_0_ is defined; λ_0_ describes the axial decay of the membrane voltage in the case of constant applied electric field. In TMS, however, the induced electric field waveform is typically a segment of a sinusoidal wave, with characteristic frequencies of the order of several kHz.

**Objective:** To show that the high frequency content of the stimulation pulse causes deviations in the spatial profile of the membrane voltage as compared to the steady state.

**Methods:** We derive the cable equation in complex form utilizing the complex frequency-dependent representation of the membrane conductivity. In addition, we define an effective length constant λ_eff_, which governs the spatial decay of the membrane voltage. We model the behavior of a dendrite in an applied electric field oscillating at 3.9 kHz with the complex cable equation and by solving the traditional cable equation numerically.

**Results:** The effective length constant decreases as a function of frequency. For a model dendrite or axon, for which λ_0_ = 1.5 mm, the effective length constant at 3.9 kHz is decreased by a factor 10 to 0.13 mm.

**Conclusion:** The frequency dependency of the neuronal length constant has to be taken into account when predicting the spatial behavior of the membrane voltage as a response to TMS.

## 1. Introduction

In transcranial magnetic stimulation (TMS; Barker et al., 1985), strong, rapidly changing magnetic-field pulses are delivered to the brain in order to induce an electric field at the target site. The electric field, in turn, produces electric currents that, when directed through cell membranes, change transmembrane potentials. If the membranes become sufficiently depolarized, action potentials are triggered; this neuronal activation is the primary outcome of TMS.

Typically, the magnetic field is produced with a coil wound in a figure-of-eight form; if such a coil is placed tangentially over the scalp, a current pulse in the coil induces a reasonably focal electric field in the superficial brain. The primary electric field is very homogeneous on the cellular scale; however, the complicated conductivity structure of the neurons, to a large extent defined by cell membranes, changes the local electric current patterns dramatically. Because the microscopic tissue structure is not generally available, precise activation patterns usually cannot be predicted; one has to rely on computing the transmembrane potentials in assumed, generally highly simplified, neuronal geometries. Often, the cable equation (e.g., Roth and Basser, 1990; Nagarajan et al., 1993; Nagarajan and Durand, 1996) is used.

The cable equation, as described in Section 2.1, describes a passive axon or dendrite as a cylinder defined by a resistive–capacitive membrane and conducting intracellular fluid. The analysis leads to two useful concepts, the length constant and the membrane time constant. The length constant describes the rate of exponential decay of membrane voltage as a function of distance from the location where current is injected (typically, a synapse or site of transmembrane ion flow during an action potential). The classical length constant is defined in the limit of low frequencies or for dc currents. The time constant describes the exponential decay of membrane capacitance via current leakage through the resistive membrane. This leakage has been assumed to weaken the effect of TMS when long-risetime pulses are used.

Here, we also use the cable equation but define the length constant for alternating currents instead of dc currents. It turns out that at the characteristic frequencies in TMS, i.e., several kHz, the length constant is far shorter than at dc. This has consequences on how neurons are activated since the area of the the membrane that is depolarized is proportional to the length constant.

## 2. Methods

### 2.1. Cable equation

**Figure 1:**
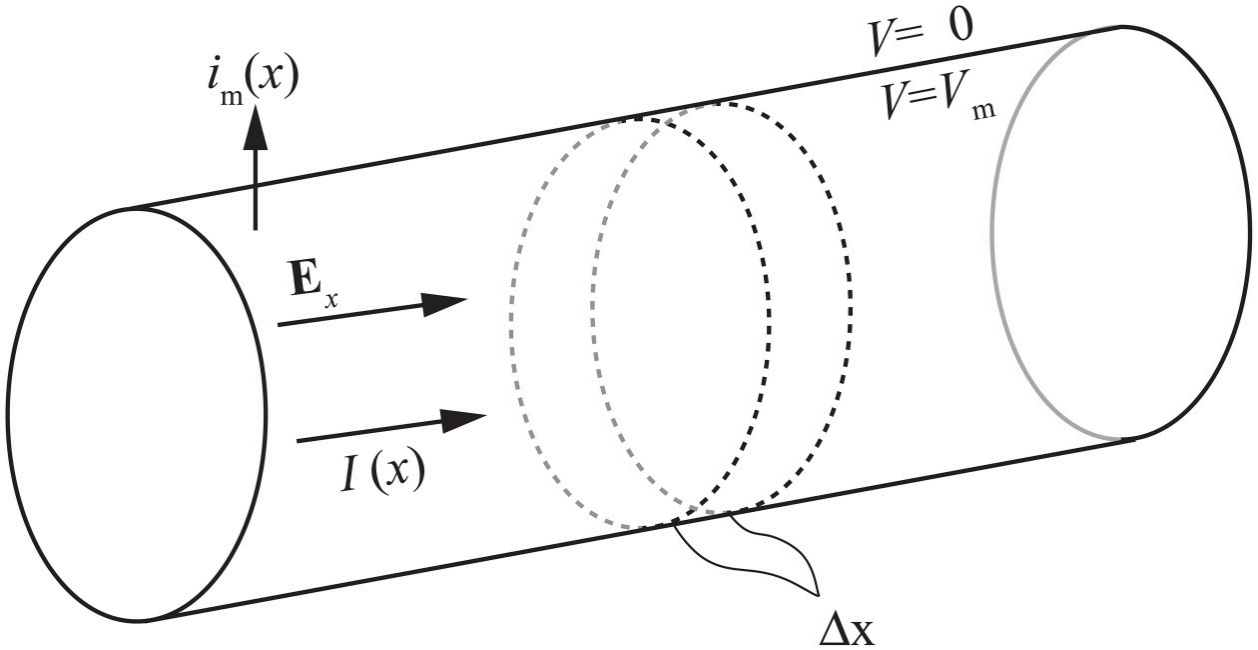
**The cylindrical cell model used for deriving the cable equation**.

In case of a passive cell, the cable equation is derived as follows:

The axial current *I*_*i*_ inside the cell (see Figure 1) is given by

(1) $$\begin{array}{r} I_{i}\left(x,t\right)=-\frac{1}{r_{i}}\frac{\partial V_{m}\left(x,t\right)}{\partial x}+\frac{1}{r_{i}}E_{x}\left(x,t\right), \end{array}$$

where *x* is the position along the longitudinal axis of the cell, *V*_*m*_ is the membrane potential, which equals the potential inside the cell if the extracellular potential is zero, *E*_*x*_ is the axial component of the applied electric field, and *r*_*i*_ is the axial resistance of the cytoplasm per unit length. The law of conservation of currents gives the membrane current per unit length

(2) $$\begin{array}{r} i_{m}\left(x,t\right)=-\frac{\partial I_{i}\left(x,t\right)}{\partial x}=\frac{1}{r_{i}}\frac{\partial^{2}V_{m}\left(x,t\right)}{\partial x^{2}}-\frac{1}{r_{i}}\frac{\partial E_{x}\left(x,t\right)}{\partial x}, \end{array}$$

which, in case of a spatially constant applied electric field over the length of the axon (∂*E*_*x*_(*x, t*)/∂*x* = 0), reduces to

(3) $$\begin{array}{r} i_{m}\left(x,t\right)=\frac{1}{r_{i}}\frac{\partial^{2}V_{m}\left(x,t\right)}{\partial x^{2}}. \end{array}$$

The membrane current can be presented also as the sum of ohmic and capacitive components

(4) $$\begin{array}{r} i_{m}\left(x,t\right)=c_{m}\frac{\partial V_{m}\left(x,t\right)}{\partial t}+\frac{V_{m}\left(x,t\right)}{r_{m}}, \end{array}$$

where *c*_*m*_ is the membrane capacitance per unit length of the cell and *r*_*m*_ is the membrane resistance of a segment of the axon times the length of the segment.

Combining Equations (3) and (4) gives the cable equation

(5) $$\begin{array}{r} \lambda_{0}^{2}\frac{\partial^{2}V_{m}\left(x,t\right)}{\partial x^{2}}=\tau \frac{\partial V_{m}\left(x,t\right)}{\partial t}+V_{m}\left(x,t\right), \end{array}$$

where

(6) $$\begin{array}{r} \lambda_{0}=\sqrt{r_{m}/r_{i}} \end{array}$$

is the steady-state length constant describing the exponential decay of the membrane potential as a function of *x* and

(7) $$\begin{array}{r} \tau =r_{m}c_{m} \end{array}$$

is the time constant of the membrane.

However, as we have documented earlier (Saari, 2009) and as was pointed out also by Meffin and Kameneva (2011), λ_0_ describes the spatial decay of membrane potential only in the steady state. This results from the fact that the cellular membrane has capacitive properties and its conductivity can be described as a frequency-dependent complex value. Although the quasi-static approximation is valid at the macroscopic level at the dominant TMS-pulse frequencies of several kilohertz (Roth et al., 1991), considerable deviations from the steady-state spatial patterns of current flow at the cellular level can be expected when TMS is applied.

In the present work, we study the frequency-dependent behavior of a neuron and formulate the effective length constant that governs the spatial decay of the membrane voltage. Preliminary results of this work have been reported in abstract form (Ilmoniemi et al., 2011).

### 2.2. Frequency-dependent membrane conductivity and neuronal length constant

Let us assume that the electric field component parallel to the neuronal axis (*E*_*x*_) is constant along the length of the axon or dendrite and oscillating at angular frequency ω:

(8) $$\begin{array}{r} E_{x}\left(t\right)=E_{0}e^{i\omega t}, \end{array}$$

which makes the membrane voltage oscillate at the same frequency:

(9) $$\begin{array}{r} V_{m}\left(x,t\right)=V_{m}\left(x\right)e^{i\omega t}. \end{array}$$

We can express the membrane current per unit length with the complex frequency-dependent conductivity σ_*m*_ as

(10) $$\begin{array}{r} i_{m}\left(x\right)=\sigma_{m}V_{m}\left(x\right)=\left(\frac{1}{r_{m}}+i\omega c_{m}\right)V_{m}\left(x\right), \end{array}$$

where *i* is the imaginary unit.

Combining Equations (10) and (3) gives a complex frequency-dependent cable equation

(11) $$\begin{array}{r} V_{m}\left(x\right)=\lambda_{f}^{2}\frac{\partial^{2}V_{m}\left(x\right)}{\partial x^{2}}, \end{array}$$

where

(12) $$\begin{array}{r} \lambda_{f}=\lambda_{f}\left(\omega \right)=\sqrt{\frac{1}{\frac{r_{i}}{r_{m}}+i\omega c_{m}r_{i}}}. \end{array}$$

In case of a semi-infinite cable with the end at *x* = 0, the solution of Equation (11) is

(13) $$\begin{array}{r} V_{m}\left(x\right)=De^{-x/\lambda_{f}}, \end{array}$$

where *D* is a constant that depends on the boundary condition. At zero frequency (ω = 0), λ_*f*_ = λ_0_, the steady-state length constant.

Equation (13) can be divided into real and imaginary parts:

(14) $$\begin{array}{l} V_{\text{m}}(x)=\left(\text{Re}\left(D\right)+i\text{ Im}\left(D\right)\right)\left({\text{e}}^{-\text{Re}\left(1/\lambda_{\text{f}}\right)x-i\text{ Im}\left(1/\lambda_{\text{f}}\right)x}\right)\\ \;={\text{e}}^{-\text{Re}\left(1/\lambda_{\text{f}}\right)x}[\{\text{Re}\left(D\right)+i\text{ Im}\left(D\right)\}\{\text{cos}\left(cx\right)\\ \;+\;i\text{ sin}\left(cx\right)\}], \end{array}$$

where *c* = −*Im*(1/λ_*f*_).

From Equation (14), we see that the real behavior of the membrane voltage can be expressed as:

(15) $$\begin{array}{l} \text{Re}\left(V_{\text{m}}(x)\right)={\text{e}}^{-\text{Re}\left(1/\lambda_{\text{f}}\right)x}\left[\text{Re}\left(D\right)\text{cos}\left(cx\right)-\text{Im}\left(D\right)\text{sin}\left(cx\right)\right]\\ \;={\text{e}}^{-x/\lambda_{\text{eff}}}\left[\text{Re}\left(D\right)\text{cos}\left(cx\right)-\text{Im}\left(D\right)\text{sin}\left(cx\right)\right], \end{array}$$

where λ_*eff*_ is the effective length constant:

(16) $$\begin{array}{r} \lambda_{e\mathrm{ff}}=\frac{1}{Re\left(1/\lambda_{f}\right)}. \end{array}$$

Substituting Equation (13) into Equation (9) gives the membrane voltage as a function of *x* and time:

(17) $$\begin{array}{r} V_{m}\left(x,t\right)=De^{-x/\lambda_{f}}e^{i\omega t}, \end{array}$$

which can also be expressed as

(18) $$\begin{array}{r} V_{m}\left(x,t\right)=e^{-x/\lambda_{eff}}\left[Re\left(D\right)e^{i\omega t+cx}-Im\left(D\right)e^{i\omega t+cx-\pi /2}\right], \end{array}$$

the real part of which is

(19) $$\begin{array}{l} \text{Re}\left(V_{\text{m}}(x,t)\right)={\text{e}}^{-x/\lambda_{\text{eff}}}[\text{Re}(D)\cos(\omega t+cx)\\ \;-\text{Im}(D)\sin\left(\omega t+cx\right)] \end{array}$$

For an applied electric field of arbitrary periodic waveform, which can be presented as a Fourier series

(20) $$\begin{array}{r} E\left(t\right)=\overset{\infty }{\underset{k=0}{\sum }}c_{k}e^{i\left(\omega_{k}t+\phi_{k}\right)}, \end{array}$$

where *c*_*k*_ and ϕ_*k*_ are the complex Fourier coefficient and phase of frequency component ω_*k*_, respectively, the membrane voltage can be written as

(21) $$\begin{array}{r} V_{m}\left(x,t\right)=\overset{\infty }{\underset{k=0}{\sum }}D\left(\omega_{k}\right)e^{-x/\lambda_{f}\left(\omega_{k}\right)}c_{k}e^{i\left(\omega_{k}t+\phi_{k}\right)}. \end{array}$$

### 2.3. Boundary condition

To solve the constant *D* in Equations (13–15) and (17–19), let us consider a leaky end boundary condition. The axial current at the end of the cell (see Equation 1) equals the current flowing into the cell through the end (see Equation 4):

(22) $$\begin{array}{r} I_{end}\left(x=0\right)=-\frac{1}{r_{i}}\frac{\partial V_{m}\left(x=0\right)}{\partial x}+\frac{1}{r_{i}}E_{x}\\ =-\left(\frac{1}{R_{end}}+i\omega C_{end}\right)V_{m}\left(x=0\right), \end{array}$$

where *R*_end_ and *C*_end_ are the resistance and capacitance of the end of the cable. Substituting Equation (13) into Equation (22) gives

(23) $$\begin{array}{r} D=\frac{E_{0}}{r_{i}/R_{end}+ir_{i}\omega C_{end}-1/\lambda_{f}} \end{array}$$

(24) $$\begin{array}{r} =E_{0}\frac{a-ib}{a^{2}+b^{2}}, \end{array}$$

where

(25) $$\begin{array}{r} a=\frac{r_{i}}{R_{end}}-Re\left(\frac{1}{\lambda_{f}}\right) \end{array}$$

and

(26) $$\begin{array}{r} b=r_{i}\omega C_{end}-Im\left(\frac{1}{\lambda_{f}}\right). \end{array}$$

### 2.4. Numerical calculations

The variation of the neuronal membrane potential when influenced by the electric field induced in TMS can also be determined by numerically solving a discretized version of the cable equation, Equation (5) (Nagarajan et al., 1993).

That was done by employing a similar approach to the one described in a previous study (Salvador et al., 2011). We started by creating compartmentalized models of neurons comprising a myelinated axon, axon hillock, initial segment, soma and an apical dendrite. The axon, axon hillock and initial segment were modeled with an active membrane model (Wesselink et al., 1999). The soma and apical dendrites were modeled as passive compartments (RC circuits). We then specified a uniform electric field along a direction parallel to the apical dendrite. At both ends of the neuron, sealed-end boundary conditions were enforced (Nagarajan et al., 1993). Since the area of the end of a dendrite is very small, the difference between sealed-end and leaking-end boundary conditions (as used in Section 2.3) is negligible.

The resulting set of non-linear equations was solved using the Crank-Nicholson method, with a staggered time step approach (Hines, 1984). This algorithm was implemented in Matlab (version 2010a, www.mathworks.com). This algorithm has been validated by comparison with Neuron simulation environment (www.neuron.yale.edu/neuron/) as described in Salvador et al. (2011).

The apical dendrite was represented as a 6-mm-long cylinder with properties described in Table 1 and a resting membrane voltage of −84 mV. The dendrite was divided into 1000 cylinders each with a length of 6 μm. The cylindrical shape of the simulated apical dendrite makes the simulation results directly comparable with our analytical results.

**Table 1:** **Properties of the model dendrite**.

Radius (*r*)	4 μm
Axoplasmic resistivity (ρi)	0.33 Ωm
Ohmic membrane conductance per unit area (*G*_m_)	2.73 S/m^2^
Membrane capacitance per unit area (*C*_m_)	0.028 F/m^2^

**Figure 2:**
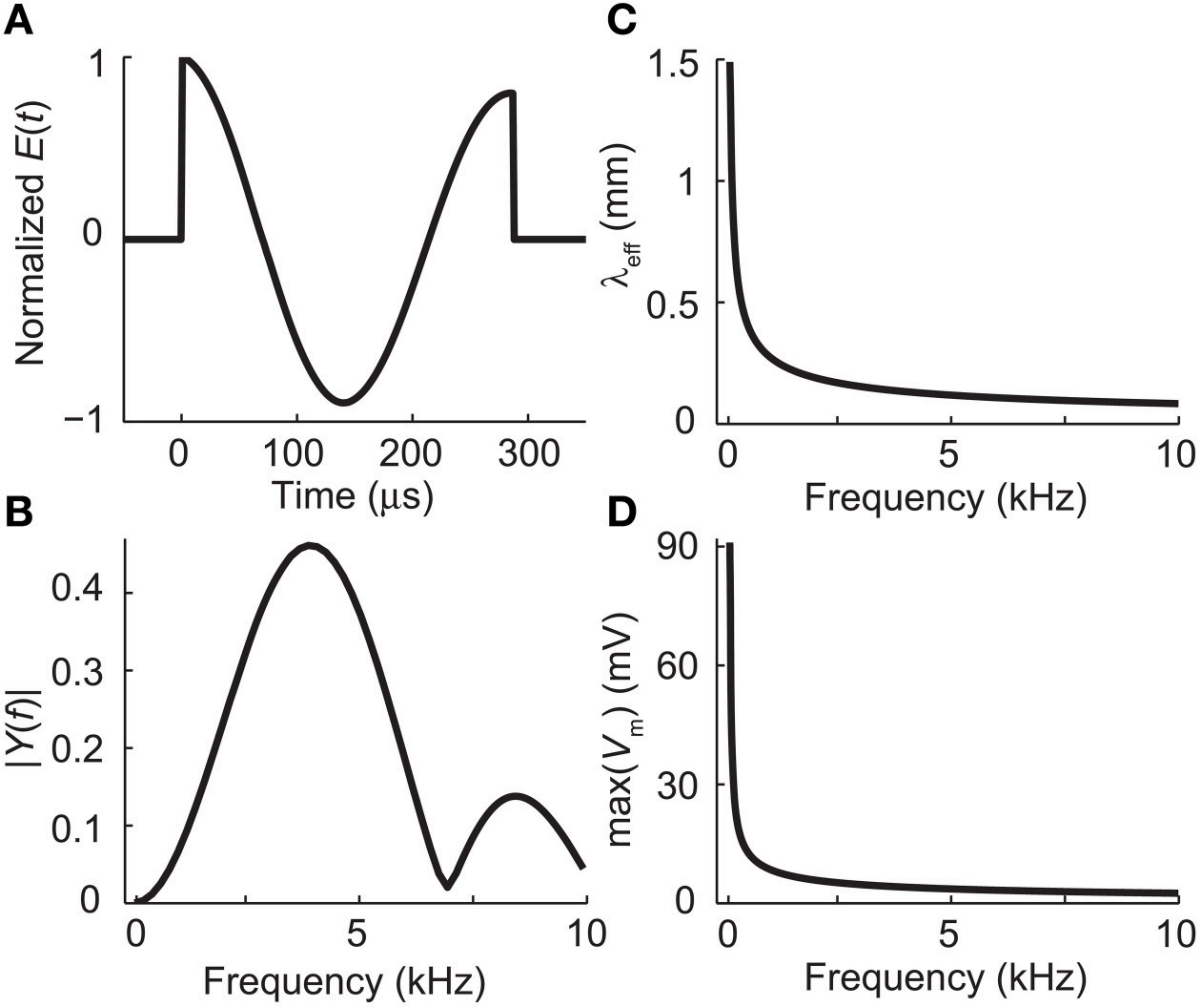
**(A)** The waveform of the electric field induced by the biphasic stimulation pulse of the Magstim Rapid stimulator. **(B)** Absolute values of the Fourier coefficients of the waveform in panel **(A)**. The waveform, sampled at 794 kHz, was zero-padded to 4096 samples to increase the apparent frequency resolution before applying the fast Fourier transform algorithm. **(C)** The effective length constant as a function of frequency, obtained from Equation (12). **(D)** The maximal deviation from the resting membrane voltage at the end of the dendrite as a function of stimulation frequency with *E*_0_ = 61.2 V/m, obtained from Equations (15) and (23).

The temporal waveform of the electric field along the neuron was sinusoidal with frequency *f* = ω/2π = 3.9 kHz and duration of 6 ms. This frequency corresponds to the peak frequency of the power-spectrum of the *dI*/*dt* pulse of the Magstim Rapid stimulator (Figures 2A,B). A time step of 1.5 μs was used in the simulations. The amplitude of the waveform was adjusted so that the applied electric field along the neuron was of 61.2 V/m (a value below stimulation threshold).

All simulations took less than a minute to solve in a computer with a quad-core CPU clocked at 2 GHz and 8 Gb of RAM.

The length constant of the numerically calculated membrane voltage was estimated with exponential curve fitting in Matlab: nonlinear least-squares fitting with the trust-region algorithm was applied to the membrane voltage curve near the end of the dendrite.

## 3. Results

For the model dendrite with properties described in Table 1, which are connected to *r*_*i*_, *r*_*m*_, and *c*_*m*_ in the following way:

(27) $$\begin{array}{r} r_{i}=\frac{\rho_{i}}{\pi r^{2}}, \end{array}$$

(28) $$\begin{array}{r} r_{m}=\frac{1}{2\pi r\;G_{m}}, \end{array}$$

(29) $$\begin{array}{r} c_{m}=2\pi r\;C_{m}, \end{array}$$

the steady-state length constant is

(30) $$\lambda_{0}=\sqrt{\frac{r_{\text{m}}}{r_{\text{i}}}}\approx 1.5\text{ mm}.$$

To show how the length constant is affected by the high-frequency stimulation, we approximate the TMS pulse as sine wave of 3.9 kHz, which is the peak frequency of the biphasic stimulation pulse of the Magstim Rapid stimulator (Figures 2A,B). The effective frequency-dependent length constant (Equation 16) at *f* = 3.9 kHz is

(31) $$\lambda_{\text{eff}}=\frac{1}{\text{Re}\left(1/\lambda_{\text{f}}\right)}=\frac{1}{\text{Re}\left(\sqrt{\frac{r_{\text{i}}}{r_{\text{m}}}+i\omega c_{\text{m}}r_{\text{i}}}\right)}\approx 0.13\text{ mm}.$$

**Figure 3:**
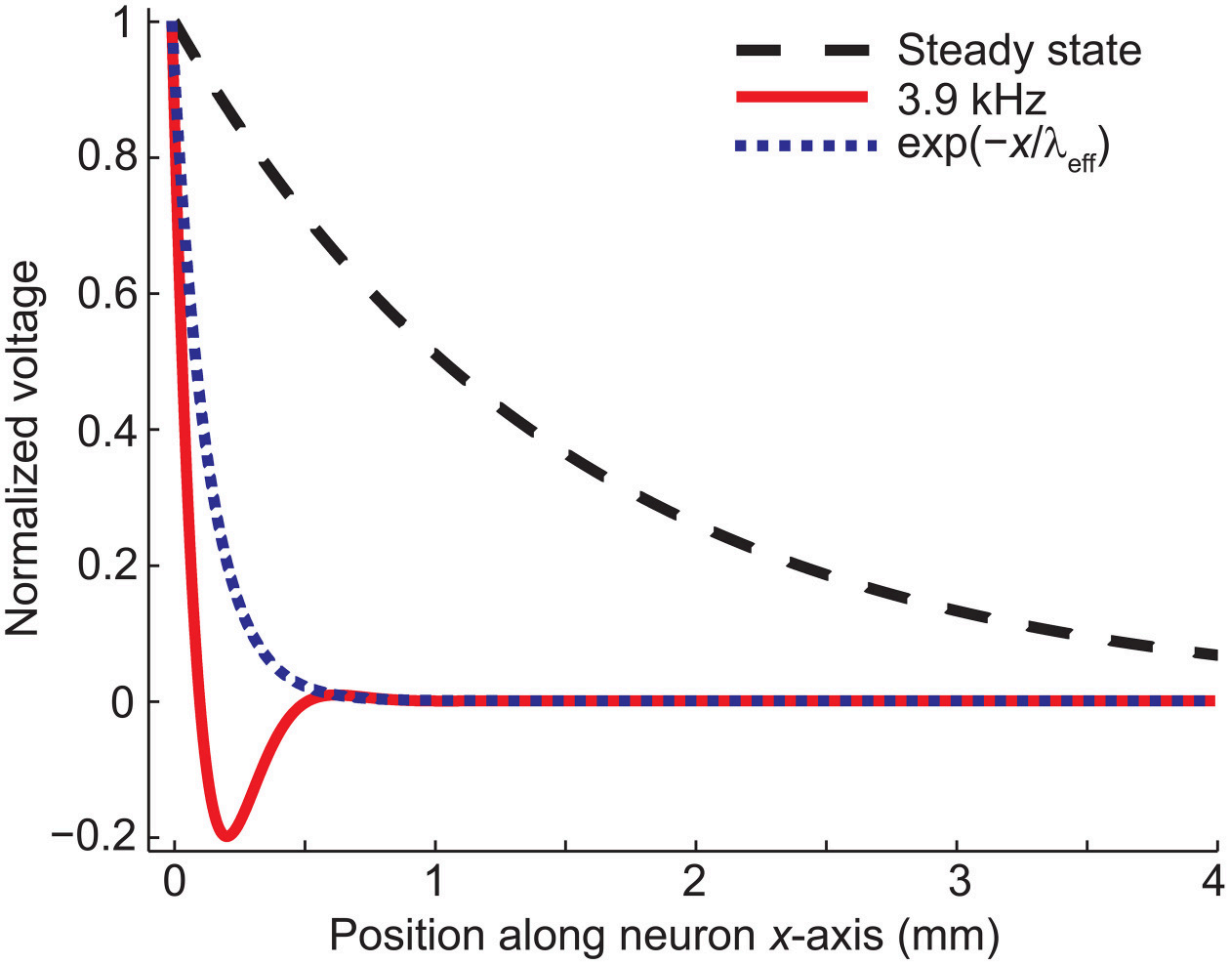
**The spatial decay of the membrane voltage in the steady state and in case of 3.9-kHz stimulation obtained from Equation (15)**. The exponential term in Equation (15) is shown for comparison. Note that the profile of the membrane potential is time-dependent due to the trigonometric term in Equation (19).

Figure 2C shows how the effective length constant (Equation 16) decreases when the stimulation frequency increases. In addition, we see from Equations (15) and (23) that the maximal membrane voltage at the end of the cell decreases as a function of frequency (Figure 2D). Figure 3 illustrates the spatial decay of the membrane potential in the steady state and with 3.9-kHz stimulation obtained from Equation (15).

The numerical results agreed with the analytical ones: The decay of the membrane voltage at the end of the dendrite (0…0.23 mm) at the time of maximal *E*(*t*) (*t* = 5.7555 ms) was explained (*R*^2^ = 1) with two exponentials, one with a length constant of 0.13 mm [corresponding to the exponential part of Equation (19)] and the other one with a length constant of 4.9 mm [corresponding to the sinusoidal part of Equation (19) and the DC component, i.e., resting membrane voltage].

## 4. Discussion

We have shown how the neuronal length constant decreases with increasing frequency of the stimulation pulse waveform. In TMS, the applied electric field typically has characteristic frequencies of the order of kilohertz, in which case the length constant can be an order of magnitude smaller than the steady-state value. As a consequence, the segment of a neuron where the TMS pulse can trigger voltage-dependent sodium channels is much narrower than one might have previously thought, influencing the efficacy of the initial inflow of Na^+^ current in initiating the action potential. This has potentially two major consequences. First, the threshold voltage of sodium channels is reached with less transferred charge with short than with longer pulses; thus, shorter pulses are more energy-efficient (Barker et al., 1991). On the other hand, the efficacy of stimulation might be somewhat reduced if only a very narrow neuronal segment is stimulated.

In our analysis, we made the simplifying assumption that the extracellular potential is zero or, equivalently, that the extracellular resistivity is vanishingly small. In the brain, however, the extracellular resistance per unit length cannot be assumed much smaller than the intracellular resistance per unit length. Thus, the extracellular potential is nonzero (Equation 1); qualitative understanding of TMS-induced effects, however, remains unchanged even if the extracellular potential is taken into account (Nagarajan and Durand, 1996).

One should note that the traditional cable equation (Equation 5) is also correct (which is why the numerical and analytical results coincide). The frequency dependency of the length constant can be seen by Fourier-transforming the traditional cable equation. This is what Meffin and Kameneva (2011) did. They, however, only calculated an approximation of the effective length constant. Their result is in agreement with ours.

## Author contributions

All authors listed, have made substantial, direct and intellectual contribution to the work, and approved it for publication.

## Funding

RI, HM, and JS were supported by grants from the Academy of Finland (decision numbers 121167, 256525, and 283105). RS and PM were supported in part by the Foundation for Science and Technology (FCT), Portugal, via FCTIBEB Strategic Project UID/BIO/00645/2013.

### Conflict of interest statement

The authors declare that the research was conducted in the absence of any commercial or financial relationships that could be construed as a potential conflict of interest.
